# Regulation of Adaptive Immunity; The Role of Interleukin-10

**DOI:** 10.3389/fimmu.2013.00129

**Published:** 2013-05-31

**Authors:** T. H. Sky Ng, Graham J. Britton, Elaine V. Hill, Johan Verhagen, Bronwen R. Burton, David C. Wraith

**Affiliations:** School of Cellular and Molecular Medicine, University of Bristol, Bristol, UK

**Keywords:** allergy, autoimmunity, cytokines, immune regulation, immunotherapy, interleukin-10, regulatory T cells, T helper cells

## Abstract

Since the discovery of interleukin-10 (IL-10) in the 1980s, a large body of work has led to its recognition as a pleiotropic immunomodulatory cytokine that affects both the innate and adaptive immune systems. IL-10 is produced by a wide range of cell types, but for the purposes of this review we shall focus on IL-10 secreted by CD4^+^ T cells. Here we describe the importance of IL-10 as a mediator of suppression used by both FoxP3^+^ and FoxP3^−^ T regulatory cells. Moreover, we discuss the molecular events leading to the induction of IL-10 secretion in T helper cell subsets, where it acts as a pivotal negative feedback mechanism. Finally we discuss how a greater understanding of this principle has allowed for the design of more efficient, antigen-specific immunotherapy strategies to exploit this natural phenomenon clinically.

## The Importance of IL-10

Interleukin (IL)-10 is a pleiotropic, immunoregulatory cytokine that is important in protecting the host from infection-associated immunopathology, autoimmunity, and allergy. IL-10 was initially characterized as a T helper (T_H_)2 specific cytokine (Fiorentino et al., 1989); however, further investigations revealed that IL-10 production was also associated with T regulatory (Treg) cell responses (Moore et al., 2001; O’Garra and Vieira, 2004; Roncarolo et al., 2006; Sabatos-Peyton et al., 2010). It is now known that almost all cells of both the innate and adaptive arms of the immune system can express IL-10, including dendritic cells (DC), macrophages, mast cells, natural killer cells (NK), eosinophils, neutrophils, B cells, CD8^+^ T cells, and T_H_1, T_H_2, and T_H_17 CD4^+^ T cells (Maloy and Powrie, 2001; Moore et al., 2001; Fillatreau et al., 2002; Roncarolo et al., 2006; O’Garra and Vieira, 2007; Trinchieri, 2007; Maynard and Weaver, 2008; Sabatos-Peyton et al., 2010; Mauri and Bosma, 2012). For the purposes of this review, we will focus on the expression of IL-10 by CD4^+^ T cells and how it acts upon T_H_ cells to promote immune homeostasis.

The first IL-10-deficient mouse model was reported 20 years ago and in the past two decades a great deal has been learned about the complex biology of IL-10 by studying this model (Kühn et al., 1993). IL-10-deficient mice exhibit prolonged and exaggerated immune responses toward antigen, in many cases accompanied by excessive inflammation and tissue damage, and they often develop chronic enterocolitis (Kühn et al., 1993; Leon et al., 1998). This pathology is ameliorated under germ-free conditions, suggesting a role for the gut flora in triggering disease and, therefore, a role for IL-10 in regulating homeostatic interactions with commensal microorganisms (Sellon et al., 1998). Similarly, IL-10-deficient mice develop prolonged and exacerbated fever in response to lipopolysaccharide (LPS) (Leon et al., 1998) and suffer a lethal immune response to acute infection with *Toxoplasma gondii*, which is not seen in wildtype animals (Gazzinelli et al., 1996). IL-10-deficiency also aggravates autoimmune pathology in a range of experimental models including rheumatoid arthritis (RA) (Hata et al., 2004), experimental autoimmune neuritis (Bai et al., 1997), systemic lupus erythematosus (SLE) (Beebe et al., 2002), and experimental autoimmune encephalomyelitis (EAE) (Bettelli et al., 1998).

Several studies of human autoimmune disease have revealed that the level of IL-10 detected in patient samples correlates inversely with disease severity (Hajeer et al., 1998; Lim et al., 1998; Crawley et al., 1999; Van Boxel-Dezaire et al., 1999; Gibson et al., 2001). In multiple sclerosis (MS) patients, low levels of IL-10 mRNA in peripheral blood monocytes (PBMC) are associated with relapse and with secondary progressive disease (Van Boxel-Dezaire et al., 1999). In juvenile onset arthritis, a single-nucleotide polymorphism (SNP) associated with reduced IL-10 mRNA expression correlates with arthritis occurring in a higher number of joints (Crawley et al., 1999). SNPs associated with lower IL-10 mRNA expression are also overrepresented in patients with RA (Hajeer et al., 1998), severe asthma (Lim et al., 1998), and SLE (Gibson et al., 2001).

Together, these studies in mouse and man demonstrate the importance of IL-10 in immune regulation and the impact of IL-10 dysregulation on a wide range of disease states.

## Thymically and Peripherally Generated FoxP3^+^ Regulatory T Cells Secrete IL-10

Regulatory T cells expressing the master transcription factor forkhead box P3 (FoxP3) are essential for immune homeostasis (Chaudhry and Rudensky, 2013). Loss of function mutations within the *Foxp3* locus result in congenital Treg deficiency and severe systemic immunopathology in both man (Gambineri et al., 2003) and mouse (Brunkow et al., 2001). Natural, or thymic, Foxp3^+^ Tregs (tTreg) develop during selection against self-antigen in the thymus (Fontenot et al., 2003), whereas peripherally induced Foxp3^+^ Tregs (pTreg) develop extrathymically in response to antigen-specific stimulation in the presence of transforming growth factor beta (TGF-β) (Curotto de Lafaille et al., 2004). tTregs are implicated in tolerance to self-antigens (Hori et al., 2003), whilst pTregs appear to modulate immune responses against both self-antigens not expressed in the thymus and foreign antigens (Pacholczyk et al., 2006; Josefowicz et al., 2012; Samstein et al., 2012). Although several groups have attempted to define a phenotype which distinguishes tTreg and pTreg (Thornton et al., 2010; Weiss et al., 2012; Yadav et al., 2012), at the time of writing, no molecular markers have been identified that can adequately discriminate these two types of Foxp3^+^ Treg cells, especially under inflammatory conditions (Verhagen and Wraith, 2010; Gottschalk et al., 2012; Weiss et al., 2012; Himmel et al., 2013).

FoxP3^+^ Tregs are able to secrete IL-10 and this appears to be particularly important in regulating immune responses at the body’s environmental interfaces (Uhlig et al., 2006; Maynard et al., 2007; Rubtsov et al., 2008). Mice with selective knockout of IL-10 in Foxp3-expressing cells (IL-10^fl/fl^ × FoxP3-cre) do not develop spontaneous systemic autoimmunity but do develop spontaneous colitis in a similar manner to germline IL-10-knockout animals (Rubtsov et al., 2008). These mice also develop heightened lung inflammation following intranasal challenge with ovalbumin (OVA), characterized by increased IL-5, IL-13, and interferon-gamma (IFN-γ) mRNA in lung tissue (Rubtsov et al., 2008). IL-10^fl/fl^ × FoxP3-cre mice exhibit exacerbated skin hypersensitivity when challenged with dinitrofluorobenzene (Chang et al., 2002). The secretion of IL-10 by FoxP3^+^ Treg cells is also important in regulating immune responses against self-antigens in some animal models. In the non-obese diabetic (NOD) mouse model of Type 1 diabetes, disease progression is associated with gradual loss of pancreatic IL-10-secreting FoxP3^+^ Tregs (Kornete et al., 2012). In this model, inducible T cell costimulator (ICOS) blockade results in reduced IL-10 secretion by ICOS^+^ FoxP3^+^ Tregs and this is associated with exacerbated diabetes (Kornete et al., 2012). Regulation of murine T_H_17 responses by FoxP3^+^ Tregs is dependent upon a Treg-specific IL-10-induced transcriptional program, which includes signal transducers and activators of transcription (STAT)3 dependent induction of Treg-derived IL-10 (Chaudhry et al., 2011). Selective deletion of *IL-10RA* on FoxP3^+^ Tregs reduces their expression of IL-10 and renders them unable to prevent IL-17-mediated pathology (Chaudhry et al., 2011).

## FoxP3^−^ Regulatory T Cells Secrete IL-10

Following stimulation under specific conditions, naïve CD4^+^ T cells can differentiate into a population of FoxP3^−^, IL-10-secreting T cells with potent regulatory capacity (Groux et al., 1997). Often termed type 1 regulatory T cells (Tr1), they are characterized by the expression of high levels of IL-10, sometimes concomitant with IL-5 or IFN-γ, and low expression of IL-2 and IL-4 (Groux et al., 1997). A recent study found that Tr1 cells can be identified by CD49b and LAG-3 expression in both human and mice (Gagliani et al., 2013). Tr1 cells can be generated *in vitro* from naïve human and murine CD4^+^ T cells by various methods, including repeated T cell receptor (TCR) stimulation in the presence of high concentrations of exogenous IL-10 (Groux et al., 1997). In *in vitro* cultures, antigen-presenting cells (APC) are required to generate Tr1 cells from IL-10 treated naïve CD4^+^ T cells (Gregori et al., 2010). This suggests that IL-10 does not act directly upon naïve CD4^+^ T cells but rather upon APC to render them able to promote Tr1 induction.

In man, a subset of peripheral blood DC, termed DC-10, and characterized by secretion of relatively high amounts of IL-10 and low amounts of IL-12, are particularly able to induce the development of Tr1-like cells *in vitro* (Gregori et al., 2010). DCs with a similar phenotype to DC-10 cells can also be generated *in vitro* by culturing human monocytes with IL-4, granulocyte macrophage colony-stimulating factor (GM-CSF) and IL-10 (Gregori et al., 2010). These *in vitro*-generated DC-10-like cells have comparable function and phenotype to those isolated from peripheral blood and may provide a method for the induction of IL-10-secreting T cells for use therapeutically (Gregori et al., 2010). Expression of human leukocyte antigen (HLA)-G and a ligand for the shed extracellular portion of HLA-G, immunoglobulin-like transcript 4 (ILT-4 or LILRB2), are upregulated by IL-10 and have been used to identify a subset of tolerogenic DC in man (Allan et al., 1999; Manavalan et al., 2003; LeMaoult et al., 2004; Gregori et al., 2010). This suggests that IL-10-induced interactions between soluble HLA-G and DC-localized ILT-4, in subsets of DCs including DC-10 cells, is required to induce IL-10-secreting CD4^+^ T cells from naïve CD4^+^ T cells (Gregori et al., 2010).

In the mouse, IL-27 has been shown to induce Tr1-like cells from naïve CD4^+^ T cells *in vitro* (Awasthi et al., 2007)_._ IL-27 induces expression of c-Maf and IL-21 which, in combination with ICOS receptor ligation, act to promote Tr1 differentiation (Awasthi et al., 2007; Pot et al., 2009). In addition, IL-27 also upregulates the aryl hydrocarbon receptor (AhR) which, when ligated, synergizes with c-Maf to drive IL-10 expression (Apetoh et al., 2010). The secretion of IL-21 by Tr1-like cells can further induce c-Maf expression and, thus, may form an autocrine positive feedback loop promoting IL-10 induction (Pot et al., 2009). Murine DCs can be induced to secrete IL-27 by treatment with recombinant galectin-1. Galectin-1-treated DCs can induce Tr1-like cells *in vitro*, dampen myelin oligodendrocyte glycoprotein (MOG_35-55_)-induced EAE and antigen-specific proliferation of splenocytes (Ilarregui et al., 2009). Endogenous expression of galectin-1 in DCs is upregulated by IL-10, 1,25(OH)_2_-vitamin D3 (VitD3), and galectin-1 itself (Ilarregui et al., 2009; Cedeno-Laurent et al., 2012). Together these data highlight the importance of the APC in IL-10 induction in naïve CD4^+^ T cells in many experimental models.

In man, naïve CD4^+^ T cells can be directed to secrete IL-10, independent of APC, by co-ligation of the TCR and the complement receptor CD46 with either the native ligand, C3b, or with anti-CD46 antibodies (Kemper et al., 2003; Cardone et al., 2010). The importance of this pathway is reinforced by the observation that CD4^+^ T cells isolated from MS and RA patients demonstrate defective IL-10 secretion in response to stimulation with anti-CD3 and -CD46 antibodies (Astier et al., 2006; Cardone et al., 2010). Ligation of CD46 leads to robust phosphorylation of extracellular signal related kinase 1 and 2 (ERK1/2) which, as will be discussed in more detail below, is a prerequisite for IL-10 expression in CD4^+^ T cells (Zaffran et al., 2001). CD46 is not expressed by murine T cells, limiting the potential to study the role of CD46 *in vivo*, but transgenic expression of human CD46 in mice leads to elevated serum IL-10 levels following *Neisseria meningitides* infection (Johansson et al., 2005).

Tr1-like cells can also be induced in the absence of APC by stimulating naïve murine CD4^+^ T cells with anti-CD3 and -CD28 antibodies in the presence of VitD3, dexamethasone (Dex), and anti-IFN-γ, -IL-4 and -IL-12 antibodies (Barrat et al., 2002). Neutralization of IL-10 in the culture medium inhibits development of Tr1 cells (Barrat et al., 2002). *In vivo*, Tr1 cells can be generated by immunizing mice with cholera toxin (Lavelle et al., 2003, 2004) or treatment with rapamycin and IL-10 (Battaglia et al., 2006).

## Self-Limitation of T Helper Cells by IL-10 Secretion

Deviation from normal immune homeostasis, during infection or other insult, will result in tissue damage if allowed to persist following elimination of the target antigen. Similarly, in situations of chronic antigen exposure, such as at mucosal surfaces and in relation to self-antigen, it is essential that regulatory mechanisms exist to restore effector CD4^+^ T cell homeostasis. In addition to the regulatory effects of FoxP3^+^ and FoxP3^−^ Tregs, an IL-10-dependent negative feedback loop is important in protecting tissues from T cell-mediated autoimmune disease and infection-driven immunopathology. For example, wildtype C57BL/6 mice, when primed with MOG in complete Freund’s adjuvant (CFA), develop EAE associated with T_H_1 and T_H_17 cytokines, but recover rapidly (Bettelli et al., 1998). In contrast, IL-10-deficient mice do not recover and develop a progressive form of EAE (Bettelli et al., 1998).

## T_H_17-Derived IL-10 in the Regulation of T_H_17 Responses

T_H_17 cells are strongly implicated in many autoimmune conditions traditionally thought to be solely T_H_1-mediated (Steinman, 2007). T_H_17 cells produce IL-17A and IL-17F, which can be detected in target tissues of patients with RA, MS, and SLE (Matusevicius et al., 1999; Linden et al., 2000; Wong et al., 2000; Hashimoto et al., 2005). Experimental colitis, induced by adoptive transfer of IL-17-secreting CD4^+^ T cells to Rag-deficient mice, can be suppressed by the co-transfer of IL-10-secreting CD4^+^ T cells (Huber et al., 2011). This suppressive effect is dependent on expression of the IL-10 receptor (IL-10R) by the IL-17-secreting CD4^+^ T cells (Huber et al., 2011). This implies that IL-10 can directly attenuate the pathogenicity of IL-17-secreting CD4^+^ T cells.

**Figure 1:**
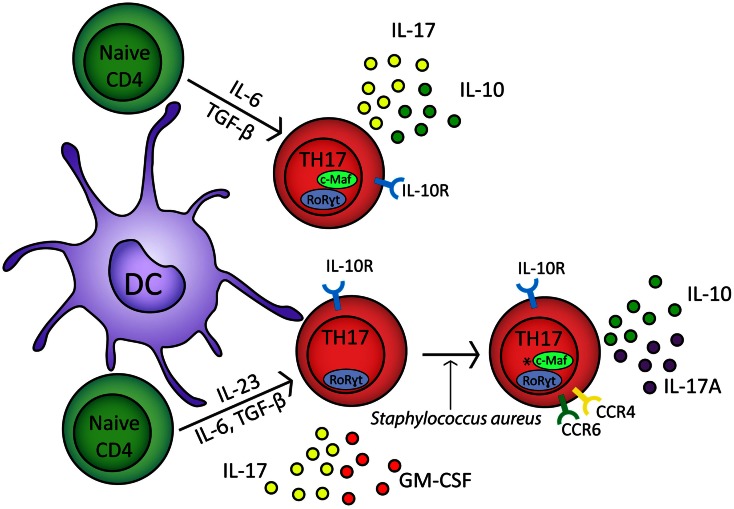
**In response to IL-6, TGF-β, and TCR stimulation naïve CD4^+^ cells upregulate RoRγt and c-Maf transcription factors and develop into T_H_17 polarized naïve CD4^+^ cells which secrete IL-17 and IL-10 (McGeachy et al., 2007)**. In the presence of IL-6, TGF-β, IL-23, and TCR stimulation, naïve CD4^+^ cells differentiate into effector T_H_17 cells which express RoRγt and secrete IL-17 and GM-CSF (McGeachy et al., 2009). In response to *Staphylococcus aureus*, effector T_H_17 cells develop into CCR6^+^ CCR4^+^ memory cells which secrete IL-17A and IL-10; *however, it is unclear if c-Maf regulates IL-10 in these cells (Metzler and Wraith, 1993; Zielinski et al., 2012). T_H_17 cells express the IL-10 receptor and can therefore self-regulate (Stumhofer et al., 2007; Huber et al., 2011). IL-1β can down regulate IL-10 expression from both T_H_17 polarized naïve CD4^+^ cells and T_H_17 memory cells (Zielinski et al., 2012).

T_H_17 cells have a developmental pathway that overlaps with FoxP3^+^ iTregs (Bettelli et al., 2006). Culturing naïve CD4^+^ T cells with TGF-β alone drives the generation of FoxP3^+^ iTregs but in combination with the inflammatory cytokine IL-6 generates T_H_17 cells which express RoRγt and secrete IL-17 (Figure 1) (Ivanov et al., 2006; McGeachy et al., 2007, 2009). In murine transfer experiments, IL-6- and TGF-β-generated T_H_17 cells failed to induce EAE; in contrast, T_H_17 cells cultured with IL-23 were encephalitogenic (Figure 1) (McGeachy et al., 2007). This correlates with enhanced expression of IL-10 in T_H_17-polarized CD4^+^ T cells cultured in the absence of IL-23 (McGeachy et al., 2007).

Interleukin-10-secreting T_H_17-polarized CD4^+^ T cells have also been observed with defined pathogen specificity. Upon restimulation *in vitro*, human *Staphylococcus aureus*-specific T_H_17 cells secreted IL-10 in combination with IL-17A (Figure 1) (Zielinski et al., 2012). In contrast, *Candida albicans*-specific T_H_17-polarized CD4^+^ T cells did not secrete IL-10 and instead secreted IFN-γ concomitant with IL-17A (Zielinski et al., 2012). IL-10 secretion by naïve CD4^+^ T cells polarized toward IL-17 secretion or by CCR6^+^CCR4^+^ memory T_H_17 cells was inhibited by IL-1β (Figure 1) (Zielinski et al., 2012). Cryopyrin associated periodic syndrome (CAPS) is an autoinflammatory disease characterized by excessive production of IL-1β (77). IL-10 production is significantly inhibited in T_H_17 cell clones from CAPS patients but *in vivo* administration of Anakinra, an IL-1R1 antagonist, restores IL-10 secretion by IL-17A^+^ T cell clones (Jacobs and Ciaccio, 2010; Zielinski et al., 2012). Together these studies suggest that IL-10 production by IL-17A-secreting CD4^+^ T cells may refine T_H_17 responses to target them toward specific pathogens. In addition, these results show that the lack of IL-10 secretion by T_H_17 cells is associated with autoinflammatory conditions, highlighting the importance of effector T cell-derived IL-10 in immune regulation.

## T_H_1-Derived IL-10 in the Regulation of T_H_1 Responses

T_H_1 cells, characterized by expression of the transcription factor T-bet and secretion of IFN-γ, play a central role in the clearance of intracellular pathogens (Romagnani, 1996). However, they are also responsible for mediating immune pathology and autoimmune disease in a number of settings. For example, the intracellular protozoan parasite *T. gondii* elicits an IL-12-dependent T_H_1 response which is important for controlling its replication in infected mice (Gazzinelli et al., 1994). In IL-10-deficient mice, this T_H_1 response is exacerbated and results in severe cytokine-associated immunopathology and mice succumb to disease even though parasitic growth is effectively restricted (Gazzinelli et al., 1996). This immune pathology is characterized by increased secretion of T_H_1 cytokines, expression of acute inflammatory markers and necrotic tissue damage (Gazzinelli et al., 1996). A dysregulated T_H_1 response and subsequent tissue damage are also observed in IL-10-deficient mice following infection with other pathogens, including *Leishmania major* (Anderson et al., 2007), *Trypanosoma cruzi* (Abrahamsohn and Coffman, 1996; Hunter et al., 1997), *Plasmodium chabaudi* (Linke et al., 1996), *Listeria monocytogene*s (Deckert et al., 2001), murine cytomegalovirus (Oakley et al., 2008), and respiratory influenza virus (Sun et al., 2009). Similarly, IL-10-dependent T_H_1 self-regulation is essential in restraining the immune response and preventing tissue damage in models of autoimmune disease including colitis (Suri-Payer and Cantor, 2001), RA (Hata et al., 2004), neuritis (Bai et al., 1997), SLE (Beebe et al., 2002), and uveoretinitis (Rizzo et al., 1998). Conversely, CD4^+^ T cells which co-secrete IFN-γ and IL-10 can be isolated from patients with chronic infections, including *Mycobacterium tuberculosis* and *Leishmania donovani* (Gerosa et al., 1999; Kemp et al., 1999; Boussiotis et al., 2000). This suggests that IL-10 secretion by T_H_1-like cells regulates the anti-pathogen response and prevents clearance.

**Figure 2:**
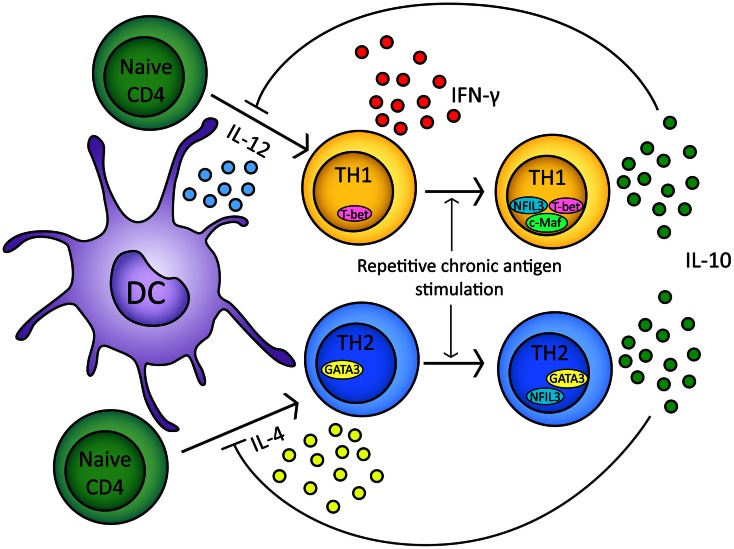
**In response to either IL-12 or IL-4 and TCR stimulation naïve CD4^+^ T cells will upregulate T-bet or GATA3 transcription factors respectively**. Differentiation of naïve CD4^+^ t cells into T_H_1/T_H_2 lineages is based on T-bet/GATA3 transcription factor expression (Ouyang et al., 2000; Lucas et al., 2003). Upon repeated chronic TCR stimulation T_H_1 and T_H_2 cells express IL-10 (Gabryšová et al., 2009; Xu et al., 2009). In T_H_1 cells, IL-10 expression is regulated by NFIL3 and correlates with c-Maf expression (Kim et al., 1999; Saraiva et al., 2009). In T_H_2 cells, IL-10 expression is regulated by NFIL3 (Motomura et al., 2011). IL-10 secreted by T_H_1/T_H_2 cells can inhibit further naïve CD4^+^ differentiation by inhibiting IL-12/IL-4 and DC function (Moore et al., 2001; Taylor et al., 2007, 2009).

Interleukin-10-secreting, T_H_1-like cells can be induced in experimental models by repeated or chronic administration of antigen (Figure 2) (Metzler and Wraith, 1993; Gabryšová et al., 2009; Gabryšová and Wraith, 2010). In the Tg4 TCR-transgenic mouse model, repeated intranasal (i.n.) administration of analogs of the Ac1-9 peptide of myelin basic protein drives the generation of FoxP3^−^ T-bet^+^ IL-10-secreting CD4^+^ T cells which protect animals from EAE (Rogge et al., 1997; Burkhart et al., 1999; Gabryšová et al., 2009). IL-10 secreted by T-bet^+^ CD4^+^ T cells modulates DC function, inducing downregulation of MHC class II, the co-stimulatory molecules CD80, CD86, and CD40, and the T_H_1-promoting cytokine IL-12 (Moore et al., 2001; Gabryšová et al., 2009). This renders DCs from Tg4 mice treated repeatedly with the Ac1-9 analog less effective than DCs from non-peptide treated mice at priming naïve CD4^+^ T cells and promoting T_H_1 differentiation (Gabryšová et al., 2009). This represents a therapeutically exploitable negative feedback loop for the attenuation of IFN-γ-driven inflammatory responses (Figure 2).

Although IL-10-mediated negative feedback regulation of CD4^+^ effector lymphocyte responses undoubtedly evolved to prevent collateral tissue damage during immune responses to pathogens, it can also prevent successful clearance of microorganisms and lead to prolonged chronic infection. For example, in mice infected with *L. major*, CD4^+^ Foxp3^−^ IL-10-secreting cells are associated with development of contained, chronic, non-healing lesions (Anderson et al., 2007). Elevated CD4^+^ T cell-derived IL-10 also correlates with an inability to effectively clear *M. tuberculosis* (Redford et al., 2011), *L. monocytogenes* (Dai et al., 1997), *Mycobacterium leprae* (Sieling et al., 1993), and transformed cells, for example squamous cell carcinomas (Kim et al., 1995).

## T_H_2-Derived IL-10 in the Regulation of T_H_2 Responses

The cytokines IL-4, IL-5, and IL-13, secreted by T_H_2 cells, provide protective immunity in the context of parasite infection (Korenaga et al., 1991; Urban et al., 1991), but also initiate, amplify, and prolong allergic responses by enhancing production of IgE and are responsible for recruitment, expansion, and differentiation of eosinophils and mast cells (Robinson et al., 1992; Romagnani, 1994; Umetsu and DeKruyff, 1997, 1999; Northrop et al., 2006). Early studies of experimental T_H_2-inducing parasitic infections, including *Trichuris muris* and *T. cruzii* demonstrated a key role for IL-10 in preventing a lethal T cell response (Silva et al., 1992; Barbosa de Oliveira et al., 1996; Schopf et al., 2002). The exaggerated cytokine response observed in IL-10-deficient mice was initially assumed to be due to a requirement for IL-10 in antagonizing deleterious T_H_1 responses (Silva et al., 1992; Barbosa de Oliveira et al., 1996; Schopf et al., 2002). More recently, it has become clear that T_H_2-derived IL-10 is also associated with downregulation of IL-4 and IL-13 during allergic responses (Grünig et al., 1997; Jutel et al., 2003; Akdis et al., 2004). In a mouse model of allergic bronchopulmonary aspergillosis, IL-10 is crucial in restraining T_H_2 responses (Grünig et al., 1997). After repeated inhalation of *Aspergillus fumigatus* allergens, lung cells and broncho-alveolar lavage (BAL) fluid from IL-10-knockout mice produced higher levels of IL-4, IL-5, and IFN-γ, leading to exaggerated airway inflammation (Grünig et al., 1997). In addition, alveolar macrophages isolated from asthmatic patients secrete lower levels of IL-10 compared to those from non-asthmatics (Borish, 1998; John et al., 1998). In healthy bee keepers, regular bee venom exposure elicits a regulatory response characterized by antigen-specific IL-10 secretion and a reduction in IL-4 and IL-13 production over the course of the bee season (Meiler et al., 2008). TGF-β appears to play a minor role in the effect and little increase in FoxP3 expression is observed after bee venom exposure, suggesting that repeatedly activated allergen-specific T cells, and not FoxP3^+^ Tregs induced *de novo*, are the source of regulatory IL-10 (Meiler et al., 2008).

In mouse models of allergy, it is clear that IL-10 plays an important role in mediating successful antigen-specific therapeutic tolerance. For example, intranasal administration of peptide derived from OVA can reduce symptoms of T_H_2-driven OVA/alum-induced airway hypersensitivity (AHR) (Akbari et al., 2001). Protection from AHR is associated with induction of IL-10-secreting pulmonary DCs with capacity to induce IL-4 and IL-10-secreting OVA-specific CD4^+^ T cells *in vitro* (Akbari et al., 2001). In addition, adoptive transfer of DCs from i.n. OVA treated mice to naïve animals induced OVA-specific CD4^+^ T cell unresponsiveness in recipients. Transfer of IL-10-deficient DCs does not induce tolerance in recipient mice (Akbari et al., 2001). Similarly, neutralization of IL-10 during tolerance induction results in elevated OVA-specific IgE production and negates the protective effect of OVA administration (Vissers et al., 2004). Successful allergen-specific immunotherapy (SIT) in man, for example in the treatment of grass pollen or house dust mite allergies, correlates with generation of IL-10-secreting CD4^+^ T cells (Francis et al., 2003; Jutel et al., 2003). IL-10 limits T_H_2 responses by downregulation of IL-4, inhibition of antigen presentation by MHC class II on DCs, and suppression of co-stimulatory molecule expression including CD28, ICOS, and CD2 (Taylor et al., 2007, 2009). This is mediated via src homology phosphatase (SHP)-1 in naïve CD4^+^ T cells, suggesting that IL-10 can regulate effector responses and also prevent the differentiation of T_H_2 cells from naïve CD4^+^ T cells (Figure 2) (Taylor et al., 2007).

## Transcriptional Regulation of IL-10 in T_H_1, T_H_2, T_H_17, and Tr1 Cells

As described above, IL-10 can be secreted by different CD4^+^ T cell types, each characterized by a distinct developmental program and hallmark transcription factors. However, some signaling pathways and transcription factors required to induce IL-10 expression are shared between CD4^+^ effector T cell subsets. The group of transcription factors regulating IL-10 transcription in all cell types has been reviewed recently (Saraiva and O’Garra, 2010) and we will focus on IL-10 transcriptional regulation in T_H_1, T_H_2, T_H_17, and Tr1 cells.

ERK1 and ERK2 activation is required for IL-10 expression in T_H_1, T_H_2, and T_H_17 cells (Saraiva et al., 2009). In CD4^+^ T cells, the strength of signaling through the TCR is proportional to ERK1 and ERK2 activation and thus to IL-10 expression (Saraiva et al., 2009). Specifically, in Th1 cells high-level TCR stimulation leads to enhanced and prolonged ERK1 and ERK2 phosphorylation which, in combination with IL-12-driven signaling through STAT4, promotes induction of IL-10 (Saraiva et al., 2009).

Recently differentiated T_H_1 cells do not secrete IL-10 and have an IL-10 promoter which is inaccessible to DNase 1 and thus not permissive for transcription (Im et al., 2004). In contrast, fully differentiated T_H_2 cells have an open, euchromatic IL-10 promoter (Im et al., 2004). In addition, histone modifications which correlate with active gene expression; histone 3 lysine 4 dimethylation (H3K4me2) and histone 4 acetylation (AcH4) are associated with the IL-10 promoter in effector T_H_2 cells but not T_H_1 cells (Im et al., 2004).

**Figure 3:**
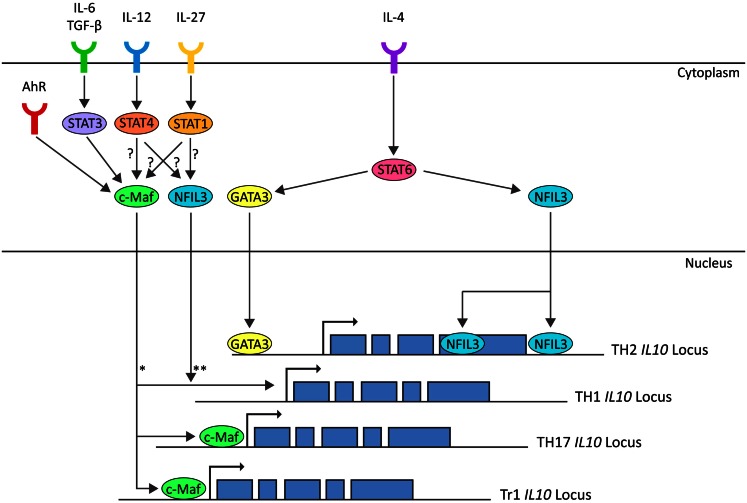
**In T_H_1 cells IL-10 expression is induced by IL-12-STAT4 and IL-27-STAT1 pathways, possibly through c-Maf and NFIL3 (Saraiva et al., 2009)**. In T_H_2 cells IL-10 expression is induced by IL-4-STAT6 through GATA3 (imprinting and chromatin modification) and NFIL3 (Shoemaker et al., 2006; Motomura et al., 2011). In T_H_17 cells IL-10 expression is induced by IL-6/TGF-β-STAT3 through c-Maf (Xu et al., 2009). In Tr1 cells, IL-10 is induced by IL-27 and AhR through induction of C-Maf (Pot et al., 2009; Apetoh et al., 2010). *c-Maf is correlated with IL-10 expression in T_H_1 cells (Saraiva et al., 2009). In non-polarizing culture conditions, IL-10 expression is dependent on c-Maf (Kim et al., 1999). Whether c-Maf binds to the IL-10 locus in T_H_1 cells is unknown. **NFIL3-deficient T_H_1 cells do not express IL-10 on repetitive stimulation, but NFIL3 has not been observed bound to the IL-10 locus in T_H_1 cells. (Motomura et al., 2011).

T_H_2 effector differentiation and function is classically described as being dependent upon STAT6-induced GATA3 expression (Shoemaker et al., 2006). Although STAT6-induced GATA3 is thought to mediate the epigenetic changes that result in an open IL-10 locus in T_H_2 cells, both proteins are dispensable for IL-10 secretion in mature T_H_2-polarized CD4^+^ T cells (Figure 3) (Ouyang et al., 2000; Shoemaker et al., 2006). Similarly, c-Maf is another transcription factor originally associated with T_H_2 cells although it is not required for IL-10 production in CD4^+^ T cells cultured under T_H_2-polarizing culture conditions (Kim et al., 1999). Interestingly, in non-polarizing culture conditions, where cells secreted IFN-γ, IL-10 production was dependent on c-Maf expression (Kim et al., 1999). Indeed, c-Maf binds to the Maf-recognition element within the IL-10 promoter and is required for IL-10 expression in Tr1, T_H_17, and possibly T_H_1 cells (Figure 3) (Kim et al., 1999; Pot et al., 2009; Saraiva et al., 2009; Xu et al., 2009). Although c-Maf is required for IL-10 secretion by a variety of CD4^+^ T cell lineages, differences in the pathways which evoke c-Maf expression reflect the diversity of the T_H_ lineages. In T_H_1 cells, IL-10 expression can be induced by both IL-27 via both STAT1 and STAT3 and IL-12 via STAT4 (Stumhofer et al., 2007; Saraiva et al., 2009). In Tr1 cells, IL-27 induces expression of c-Maf and AhR, presumably through STAT1, which cooperatively promote IL-10 expression (Figure 3) (Pot et al., 2009; Apetoh et al., 2010). In T_H_17 cells, c-Maf expression is induced by the synergistic action of TGF-β and IL-6 via STAT3 and, in contrast to the observations in T_H_1 cells, the activation of STAT1 is antagonistic for c-Maf-induced IL-10 expression in T_H_17-polarized CD4^+^ T cells (Figure 3) (Xu et al., 2009). Which pathway drives c-Maf expression may depend on specific conditions such as the affinity and dose of antigen (Saraiva et al., 2009). For example, in T_H_1 cells, IL-12-mediated STAT4 signaling only promotes IL-10 production in combination with high-level TCR stimulation (Saraiva et al., 2009) whereas at lower levels of TCR stimulation, the same stimulus induces the development of IFN-γ-secreting T_H_1 cells that do not express IL-10 (Morinobu et al., 2002; Lucas et al., 2003; Saraiva et al., 2009).

The basic leucine zipper transcription factor nuclear factor IL-3-regulated (NFIL3 or E4BP4) has recently been shown to play a role in a range of immunological processes (reviewed in Male et al., 2012). NFIL3-deficient T_H_2 cells and FoxP3^+^ Tregs are defective in IL-10 secretion and it is also required for the upregulation of IL-10 in repeatedly stimulated T_H_1 cells (Figure 3) (Chang et al., 2007; Motomura et al., 2011). NFIL3 does not bind to the *IL10* promoter, but rather to introns within the coding region of the locus (Motomura et al., 2011). In NFIL3-deficient T_H_2 cells, these regions are heterochromatic and inaccessible, suggesting that NFIL3 may play a role in remodeling the *IL10* locus to permit transcription (Motomura et al., 2011). Upregulation of NFIL3 in T_H_2 cells is dependent on IL-4 and STAT6, even upon GATA3 overexpression (Kubo and Motomura, 2012). Further work is required to understand the pathways leading to induction of NFIL3 expression in repeatedly stimulated T_H_1 cells and to confirm that NFIL3 is a universal regulator of IL-10 expression in CD4^+^ T cells.

## Clinical Applications and Future Prospects

As described above, the ratio of secreted IL-10 to the secretion of the relevant effector cytokine (IFN-γ, IL-4, or IL-17) can dictate the outcome of a polarized CD4^+^ T cell response and, therefore, the likelihood of an effective immune response and the potential for tissue damage, through hyper- or hypo-immune activation. These observations have made IL-10 an attractive therapeutic target for intervention in a wide range of human conditions including autoimmunity, cancer, and persistent infection (O’Garra et al., 2008).

Inhaled glucocorticoids are at present the treatment of choice for asthma and severe allergic conditions. In addition to the effects of corticosteroids, including dexamethosone, on IL-10 expression *in vitro*, glucocorticoid administration to asthmatic patients enhances IL-10 production concomitant with a reduction in T_H_1 and T_H_2 effector cytokines (John et al., 1998; Richards et al., 2000). Treatment with inhaled steroids is also accompanied by expansion of CD4^+^ CD25^+^ Treg populations and upregulation of *Foxp3* gene expression in CD4^+^ T cells isolated from PBMCs (Karagiannidis et al., 2004). Furthermore, failure to upregulate IL-10 in response to steroid exposure correlates with steroid resistant disease (Hawrylowicz et al., 2002; Xystrakis et al., 2006). This further illustrates the importance of steroid-induced IL-10 in the treatment of asthma and atopy.

Several pre-clinical cancer models suggest that IL-10 acts as a negative mediator of anti-tumor immunity (Halak et al., 1999; Garcia-Hernandez et al., 2002; Yang and Lattime, 2003). These are further supported by human studies in Hodgkin’s lymphoma, B cell lymphoma, melanoma, and hepatocellular carcinoma, where elevated serum IL-10 levels correlate with poor survival (Bohlen et al., 2000; Chau et al., 2000; Nemunaitis et al., 2001; Lech-Maranda et al., 2004). These observations have supported proposals that blockade of IL-10R signaling may be a beneficial adjunct therapy in the oncology clinic. However, the pleiotropic role of IL-10 has resulted in several paradoxical observations. For example, studies investigating IL-10 levels in non-small cell lung cancer observed that higher IL-10 expression correlated with better survival (Gonzalez-Aragoneses et al., 2007). In addition, overexpression of IL-10 within tumors, in murine carcinoma and melanoma models, results in loss of tumorigenicity accompanied by an enhanced lymphocyte response (Giovarelli et al., 1995; Gerard et al., 1996; Zheng et al., 1996; Adris et al., 1999). IL-10-mediated prevention of tumor growth is dependent on T cells and/or NK cells as these effects are abrogated in immunodeficient mice (Giovarelli et al., 1995; Zheng et al., 1996). In agreement with these reports, IL-10 can stimulate NK cell and alloreactive CD8^+^ T cell responses *in vitro* and *in vivo* and may have the same effect in certain cancers or subsets of patients (Groux et al., 1998, 1999; Cai et al., 1999; Micallef et al., 1999; Lauw et al., 2000).

Systemic administration of recombinant IL-10 has been trialed in patients with psoriasis and Crohn’s disease and for the alleviation of post-operative inflammation (Colombel et al., 2001; Reich et al., 2001; O’Garra et al., 2008). This has been generally tolerated at moderate doses and has provided some clinical improvement in psoriasis patients, associated with a reduction in T_H_1 cytokines (Reich et al., 2001). However, side effects including fever and headaches were observed and, in Crohn’s disease patients, IL-10 administration led to elevated serum levels of IFN-γ and no improvement in disease symptoms (Tilg et al., 2002). Simultaneous administration of IL-10 and LPS in healthy volunteers similarly led to an exaggerated T_H_1-like response compared to LPS alone (Lauw et al., 2000). This reinforced the potential for IL-10 to play a proinflammatory role, particularly at high doses, and made the use of recombinant IL-10 as a therapeutic approach unfavorable.

The failure of systemic IL-10 administration to ameliorate T_H_1-mediated pathologies highlights the importance of refinement and specificity in the design of immunomodulatory therapy. Targeting IL-10-inducing interventions to a particular anatomical site, or to cells with defined antigen specificity, may prove far more effective than non-targeted therapies. For example, although systemic administration of IL-10 can only partially ameliorate EAE symptoms in mice (Cannella et al., 1996; Nagelkerken et al., 1997), targeted expression of IL-10 in either CD2 or MHC-II-expressing cells completely abrogates disease (Bettelli et al., 1998; Cua et al., 1999). Similarly, expression of IL-10 in the central nervous system (CNS) rendered mice resistant to EAE whereas the cytokine, introduced systemically using the same expression vector, provided little benefit (Cua et al., 2001). Interestingly, orally administered IL-10, given with low-dose MBP peptide, prevented EAE (Slavin et al., 2001). Expression of IL-10 under control of the IL-2 promoter in proteolipid protein (PLP)-specific CD4^+^ T cells renders them able to both prevent and treat EAE thereby demonstrating the efficacy of antigen-specific IL-10 induction (Mathisen et al., 1997). Similarly, studies of IL-10-secreting cell-based therapies have reinforced the advantage of antigen specificity for effective immunotherapy (Barrat et al., 2002). As described above, IL-10-secreting CD4^+^ T cells can be derived from naïve T cells following *in vitro* treatment with Dex and VitD3 (Barrat et al., 2002). In theory, this would provide a source of cells that could be used therapeutically. Using OVA-specific TCR-transgenic (DO11.10) T cells, it was demonstrated that, although IL-10-secreting CD4^+^ T cells can be generated using anti-CD3 and -CD28 polyclonal stimulation, antigen-specific stimulation is required *in vivo* for IL-10-secreting cells to prevent EAE following adoptive transfer (Barrat et al., 2002). This makes antigen-SIT a very attractive approach to realize the potential of IL-10 modulation in the treatment of autoimmune diseases.

Autoantigen- and allergen-SIT aim to restore appropriate immune responses to innocuous antigens while avoiding systemic immune suppression thus preserving host-protective immunity (reviewed in Miller et al., 2007; Sabatos-Peyton et al., 2010). A variety of strategies have emerged; some attempt to induce antigen-specific FoxP3^+^ pTregs, others to induce a “switch” between T_H_1 and T_H_2-dominated immune responses or to force effector CD4^+^ T cells toward a terminally differentiated, IL-10-secreting phenotype (Miller et al., 2007; Sabatos-Peyton et al., 2010). Regardless of the cellular mechanisms underlying the SIT, successful therapies are almost always associated with an increase in specific, antigen-induced IL-10 (Miller et al., 2007; O’Garra et al., 2008; Sabatos-Peyton et al., 2010). Antigen-SIT has proven effective in many pre-clinical models of autoimmune disease, for example EAE and the NOD diabetes model (Metzler and Wraith, 1993; Brocke et al., 1996; Tian et al., 1996; Burkhart et al., 1999; Shoda et al., 2005; Gabryšová et al., 2009; Gabryšová and Wraith, 2010; Schall et al., 2012). Translation of these therapies into the clinic has shown some efficacy in treatment of MS (Warren et al., 2006), RA (Prakken et al., 2004), SLE (Muller et al., 2008), and T1D (Thrower et al., 2009; Hjorth et al., 2011; Ludvigsson et al., 2012). For example, in a phase 1 clinical trial in T1D patients using an epitope of proinsulin (C19-A3), treatment resulted in increased serum IL-10 levels and improved glycemic control in the group which received 10 μg of peptide (Thrower et al., 2009). Interestingly, a higher dose of 100 μg did not show any beneficial clinical effect and no increase in serum IL-10 (Thrower et al., 2009).

These studies demonstrate that, when appropriately designed, SIT is safe in man and has great potential in treating a wide range of autoimmune and allergic diseases. However, further research is required to determine suitable routes of administration and to refine dosing strategies. Inappropriate antigen dosing, in particular, can lead to hypersensitivity reactions or to a lack of efficacy (Bielekova et al., 2000; Kappos et al., 2000). Administration of an escalating series of antigen doses has been widely employed in the field of allergen-SIT and this approach being increasingly adopted in autoantigen-SIT (Sabatos-Peyton et al., 2010).

In many autoimmune conditions, the antigen and immunodominant epitopes are uncharacterized, and where they are characterized, epitope spreading can lead to polyantigenic responses within a single patient (Miller et al., 2007; Sabatos-Peyton et al., 2010). This provides further challenges for the successful translation of SIT from (often monoclonal, TCR-transgenic) animal models to heterogenous groups of patients. Clearly, successful translation of this approach will rely on the ability of a therapeutic strategy to induce “bystander suppression” whereby T cells specific for epitopes within antigen A are capable of suppressing the response of T cells specific for antigens B, C, D, etc. within the same tissue. The fact that IL-10 suppresses co-stimulatory molecule expression by APC explains why IL-10 treated APC can mediate bystander suppression and why strategies designed to induce IL-10 are required for effective SIT. It will also be essential to widen our understanding of the molecular mechanisms underlying successful SIT, enabling development of adjunct therapies and adjuvants to bolster efficacy, improve safety, and aid maintenance of long-term tolerance.

In conclusion, IL-10 plays an essential and highly complex role in the modulation of adaptive immune responses. The pleiotropic nature of IL-10 has made translating the potential benefit of IL-10-modulating therapies into the clinic difficult; however, strategies designed to focus IL-10 expression onto antigen-specific T cells, including SIT for allergic and autoimmune diseases, have shown promising early results.

## Conflict of Interest Statement

The authors declare that the research was conducted in the absence of any commercial or financial relationships that could be construed as a potential conflict of interest.
